# Supplementary material for: Tracing fossil-based plastics, chemicals and fertilizers production in China
Source: Nat Commun. 2024 May 8;15:3854. doi: 10.1038/s41467-024-47930-0 (PMC11078955; doi:10.1038/s41467-024-47930-0)
Supplement: Supplementary file 1 — Supplementary Information [file 41467_2024_47930_MOESM1_ESM.pdf]

Supplemental Information

for

Tracing Fossil-Based Plastics, Chemicals and Fertilizers Production in China

Meng Jiang[1,2,9], Yuheng Cao[1,9], Changgong Liu[3], Dingjiang Chen[1,4], Wenji Zhou[5], Qian Wen[6], Hejiang Yu[1], Jian Jiang[1], Yucheng Ren[1], Shanying Hu[1,4], Edgar Hertwich[2,*], Bing Zhu[4,7,8,*]

1. Department of Chemical Engineering, Tsinghua University, Beijing 100084, China
2. Industrial Ecology Programme, Department of Energy and Process Engineering, Norwegian University of Science and Technology, Trondheim 7491, Norway
3. China Petroleum & Chemical Corporation (Sinopec), Beijing, 100083, China
4. Institute for Circular Economy, Tsinghua University, Beijing 100084, China
5. School of Applied Economics, Renmin University of China, Beijing 100872, China
6. China National Petroleum & Chemical Planning Institute, Beijing 100013, China
7. The State Key Lab of Chemical Engineering, Department of Chemical Engineering, Tsinghua University, Beijing 100084, China
8. Energy, Climate, and Environment Program, International Institute for Applied Systems Analysis, Laxenburg 2361, Austria
9. These authors contributed equally: Meng Jiang, Yuheng Cao

* Correspondence and requests should be addressed to: B.Z. (email: bingzhu@tsinghua.edu.cn), or to E.H. (email: edgar.hertwich@ntnu.no)

1. Supplementary Notes
2. Supplementary Figures S1–S18
3. Supplementary Table S1-S10

Contents

1. Supplementary Figures 3
2. Supplementary Notes 16
   2.1 Modelling Chemical Flows 16
      2.1.1 Steam cracking and other oil-based processes 16
      2.1.2 Syngas process and methanol-to-olefin 18
      2.1.3 Coke, calcium carbide, and vinyl acetate production 21
   2.2 Trace Production Mix and Feedstock Intensity 23
   2.3 Carbon Emissions of Primary Chemicals Production 24
      2.3.1 Coal-based ammonia production 25
      2.3.2 Steam cracking 26
   2.4 Uncertainty Analysis 30
   2.5 Quantify Future Emission Reduction Potential to 2030 32
      2.5.1 Grid Decarbonization (Coefficient) 32
      2.5.2 Process and Other Energy Improvements Coefficient 33
      2.5.3 Production Volumes & Routes Share 33
   2.6 Other CO_2 Emission Mitigation Potential and Cases of Alternative Technologies 37
      2.6.1 CO_2 Emission Reduction Potential of Primary Chemical Production 37
      2.6.2 Case of Green Hydrogen-Coal Chemical Production (modified from[35]) 37
      2.6.3 CCUS Cases in China (modified from [37]) 37
3. Supplementary Tables 39
4. References 42

1. Supplementary Figures

Product Category	Product	Coal-based	Oil-based	Gas-based
Intermediates / other chemicals	1-Butanol	43%	57%	0%
	2-Ethylhexanol	35%	65%	0%
	Acetic Acid	87%	0%	13%
	Acetone	0%	100%	0%
	Acrylonitrile	20%	74%	6%
	Bisphenol A	14%	86%	0%
	Caprolactam	28%	68%	3%
	Cyclohexanone	21%	79%	0%
	Epoxyethane	0%	100%	0%
	Epoxypropane	0%	100%	0%
	Ethylene Glycol (EG)	26%	74%	0%
	Formaldehyde	77%	0%	23%
	Methylene Diphenyl Diisocyanate (MDI)	21%	79%	0%
	Nitric Acid	75%	0%	25%
	Phenol	21%	79%	0%
	Phthalic Anhydride	0%	100%	0%
	Polyether Polyol	1%	99%	0%
	Styrene	16%	84%	0%
	Terephthalic Acid	0%	100%	0%
	Toluene Diisocyanate (TDI)	0%	100%	0%
	Vinly Chloride	80%	20%	0%
	Vinyl Acetate	80%	6%	13%

Figure S1 Production Mix (PM) of intermediates chemicals from the feedstock. The proportions were traced by carbon elements embodied in products.

Sub-category	Product Category	Product	Coal-based	Oil-based	Gas-based
Ethylene-Related	Primary chemicals	Ethylene	2%	2%	0%
Propylene-Related	Primary chemicals	Propylene	1%	1%	0%
C4	Primary chemicals	Butene	0%	0%	0%
		Butadiene	0%	0%	0%
BTX	Primary chemicals	Benzene	0%	0%	0%
		Toluene	0%	0%	0%
		Ortho-Xylene	0%	0%	0%
		Meta-Xylene	0%	0%	0%
		Para-Xylene	0%	0%	0%
Methanol-Related	Primary chemicals	Methanol	2%	0%	2%
Ammonia-Related	Primary chemicals	Ammonia	6%	0%	6%
Calcium Carbide-Related	Primary chemicals	Calcium Carbide	0%	0%	0%
	Plastics	Acrylonitrile Butadiene Styrene (ABS)	0%	0%	0%
		Phenol Formaldehyde Resin	1%	1%	1%
		Polycarbonate	0%	0%	0%
Ethylene-Related	Plastics	Polyethylene (PE)	2%	2%	0%
Propylene-Related	Plastics	Polypropylene (PP)	2%	2%	0%
C4-related	Plastics	Polystyrene (PS)	0%	0%	0%
		Polyurethane	0%	0%	0%
		Polyvinyl Chloride (PVC)	5%	5%	0%
	Fibers / Plastics	Polyethylene Terephthalate (PET)	0%	0%	0%
	Fibers	Nylon 6	1%	0%	1%
		Polyacrylonitrile (PAN)	1%	0%	1%
	Rubbers (elastomers)	Polybutadiene	0%	0%	0%
	Rubbers (elastomers)	Styrene-Butadiene Rubber (SBR)	0%	0%	0%
		Styrene-Butadiene-Styrene (SBS)	0%	0%	0%
Ammonia-Related	Fertilizers	Ammonium Bicarbonate	6%	0%	6%
		Ammonium Chloride	6%	0%	6%
		Ammonium Sulfate	6%	0%	6%
	Fertilizers	Diammonium Phosphate	6%	0%	6%
		Monoammonium Phosphate	6%	0%	6%
		Urea	6%	0%	6%
	Fertilizers / Explosives	Ammonium Nitrate	6%	0%	6%
Methanol-Related	Fuels	Dimethyl Ether	2%	0%	2%
C4-related	Fuels	Methyl Tert-Butyl Ether (MTBE)	1%	0%	1%
	Additives	Dioctyl Phthalate	0%	0%	0%
		Polyvinyl Alcohol (PVA)	2%	1%	1%
		Urea-Formaldehyde	3%	0%	3%

Figure S2. Uncertainty ranges (±) of the PM of primary chemicals and other chemicals (plastics, fibers, rubbers, fertilizers, explosives, fuels, and additives) from the feedstock.

Sub-category	Product Category	Product	Coal-based	Oil-based	Gas-based
Propylene-Related	Intermediates / other chemicals	1-Butanol	0%	0%	0%
		2-Ethylhexanol	0%	0%	0%
Methanol-Related	Intermediates / other chemicals	Acetic Acid	1%	0%	1%
BTX-related	Intermediates / other chemicals	Acetone	0%	0%	0%
Propylene-Related	Intermediates / other chemicals	Acrylonitrile	2%	0%	2%
	Intermediates / other chemicals	Bisphenol A	0%	0%	0%
		Caprolactam	1%	0%	1%
BTX-related	Intermediates / other chemicals	Cyclohexanone	0%	0%	0%
Ethylene-Related	Intermediates / other chemicals	Epoxyethane	0%	0%	0%
Propylene-Related	Intermediates / other chemicals	Epoxypropane	0%	0%	0%
Ethylene-Related	Intermediates / other chemicals	Ethylene Glycol (EG)	0%	0%	0%
Methanol-Related	Intermediates / other chemicals	Formaldehyde	2%	0%	2%
	Intermediates / other chemicals	Methylene Diphenyl Diisocyanate (MDI)	0%	0%	0%
Ammonia-Related	Intermediates / other chemicals	Nitric Acid	6%	0%	6%
BTX-related	Intermediates / other chemicals	Phenol	0%	0%	0%
		Phthalic Anhydride	0%	0%	0%
	Intermediates / other chemicals	Polyether Polyol	0%	0%	0%
BTX-related	Intermediates / other chemicals	Styrene	0%	0%	0%
		Terephthalic Acid	0%	0%	0%
	Intermediates / other chemicals	Toluene Diisocyanate (TDI)	0%	0%	0%
Ethylene-Related	Intermediates / other chemicals	Vinly Chloride	5%	5%	0%
	Intermediates / other chemicals	Vinyl Acetate	0%	0%	1%

Figure S3. Uncertainty ranges (±) of the Production Mix (PM) of intermediates and other chemicals by feedstock.

Route	Product	Coal-based	Oil-based	Gas-based
Ethylene-Related	Ethylene	18%	82%	0%
Propylene-Related	Propylene	23%	77%	0%
C4	Butene	0%	100%	0%
	Butadiene	0%	100%	0%
BTX	Benzene	21%	79%	0%
	Toluene	0%	100%	0%
	Ortho-Xylene	0%	100%	0%
	Meta-Xylene	0%	100%	0%
	Para-Xylene	0%	100%	0%
Methanol-Related	Methanol	76%	0%	24%
Ammonia-Related	Ammonia	75%	0%	25%
Calcium Carbide-Related	Calcium Carbide	100%	0%	0%

Figure S4. Consumption Mix (CM, including import/export) of primary chemicals by feedstock. The proportions were traced by carbon elements embodied in input reactant consumption.

Product Category	Product	Coal-based	Oil-based	Gas-based
Plastics	Polyethylene (PE)	14%	86%	0%
	Polypropylene (PP)	32%	68%	0%
	Polyvinyl Chloride (PVC)	72%	28%	0%
	Polystyrene (PS)	9%	91%	0%
	Acrylonitrile Butadiene Styrene (ABS)	7%	90%	3%
	Phenol Formaldehyde Resin	44%	40%	16%
Fibers / Plastics	Polyethylene Terephthalate (PET)	4%	96%	0%
Fibers	Polyacrylonitrile (PAN)	15%	73%	12%
Rubbers (elastomers)	Polybutadiene	0%	100%	0%
	Styrene-Butadiene Rubber (SBR)	4%	96%	0%
	Styrene-Butadiene-Styrene (SBS)	5%	95%	0%
Fertilizers	Urea	75%	0%	25%
	Monoammonium Phosphate	75%	0%	25%
	Diammonium Phosphate	75%	0%	25%
	Ammonium Chloride	75%	0%	25%
	Ammonium Bicarbonate	75%	0%	25%
	Ammonium Sulfate	75%	0%	25%
Fertilizers / Explosives	Ammonium Nitrate	75%	0%	25%
Fuels	Methyl Tert-Butyl Ether (MTBE)	27%	64%	9%
	Dimethyl Ether	76%	0%	24%
Additives	Urea-Formaldehyde	75%	0%	25%
	Dioctyl Phthalate	19%	77%	4%

Figure S5. Consumption Mix (CM, including import/export) of chemicals (plastics, fibers, rubbers, fertilizers, explosives, fuels, and additives) by feedstock. The proportions were traced by carbon elements embodied in input reactant consumption.

Product Category	Product	Coal-based	Oil-based	Gas-based
Intermediates / other chemicals	Vinly Chloride	76%	24%	0%
	Ethylene Glycol (EG)	13%	87%	0%
	Epoxyethane	0%	100%	0%
	Acrylonitrile	18%	73%	9%
	Epoxypropane	0%	100%	0%
	1-Butanol	36%	57%	7%
	2-Ethylhexanol	33%	65%	3%
	Styrene	11%	89%	0%
	Cyclohexanone	21%	79%	0%
	Phenol	17%	83%	0%
	Acetone	0%	100%	0%
	Terephthalic Acid	0%	100%	0%
	Phthalic Anhydride	0%	100%	0%
	Formaldehyde	76%	0%	24%
	Acetic Acid	87%	0%	13%
	Nitric Acid	74%	0%	26%
	Polyether Polyol	1%	99%	0%
	Methylene Diphenyl Diisocyanate (MDI)	18%	82%	0%
	Toluene Diisocyanate (TDI)	0%	100%	0%
	Caprolactam	26%	70%	4%
	Bisphenol A	10%	90%	0%
	Vinyl Acetate	72%	9%	19%

Figure S6. Consumption Mix (CM, including import/export) of intermediates and other chemicals by feedstock. The proportions were traced by carbon elements embodied in input reactant consumption.

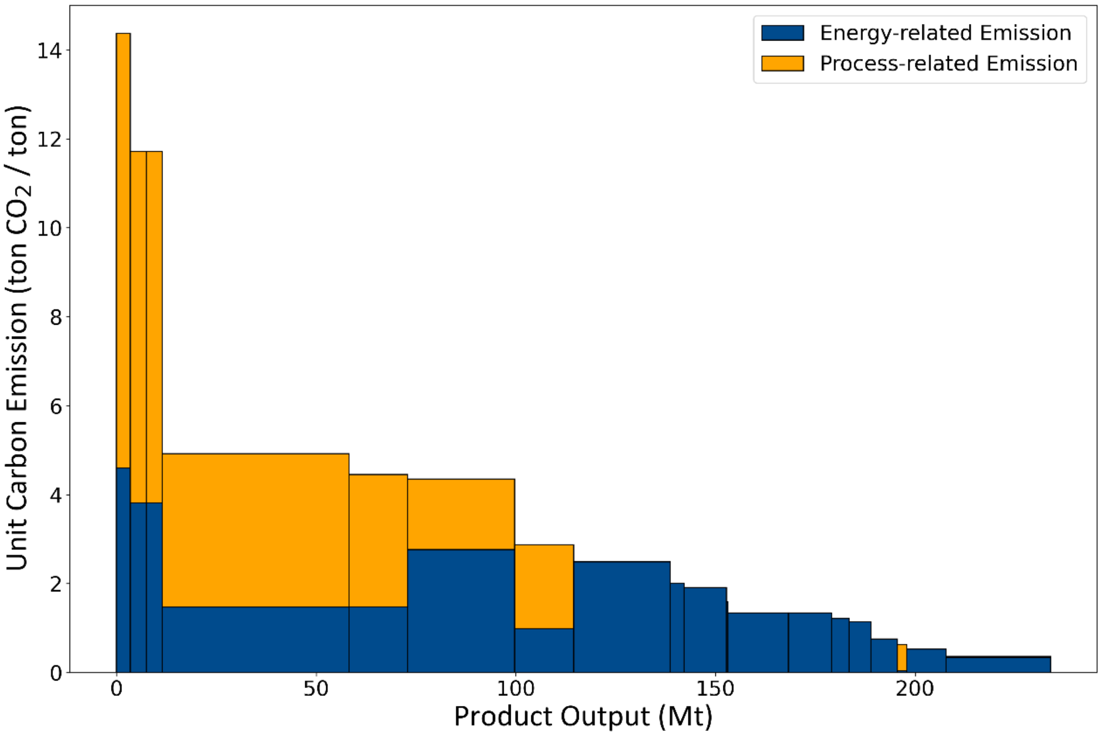

Figure S7. Process-related and Energy-related Carbon Emissions of Primary Chemicals Production (at the upper boundary of the uncertainty range). The Y-axis indicates the carbon emission per unit product (ton CO_2 per ton product). The X-axis is the output of an individual product. In this form, each rectangle area represents the total emission of production per product, differentiated by process-related emission (in yellow) and energy-related emission (in blue).

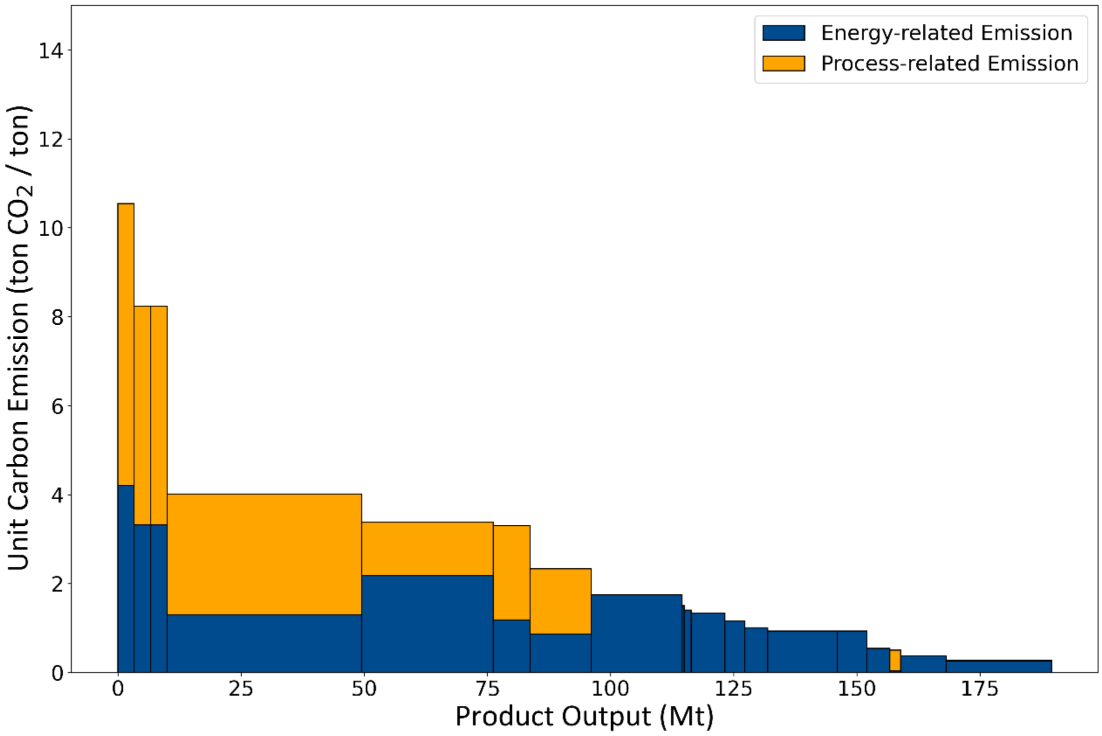

Figure S8. Process-related and Energy-related Carbon Emission of Primary Chemicals Production (at the lower boundary of uncertainty range). The Y-axis indicates the carbon emission per unit product (ton CO_2 per ton). The X-axis is the output of an individual product. In this form, each rectangle area represents the total emission of production per product, differentiated by process-related emission (in yellow) and energy-related emission (in blue).

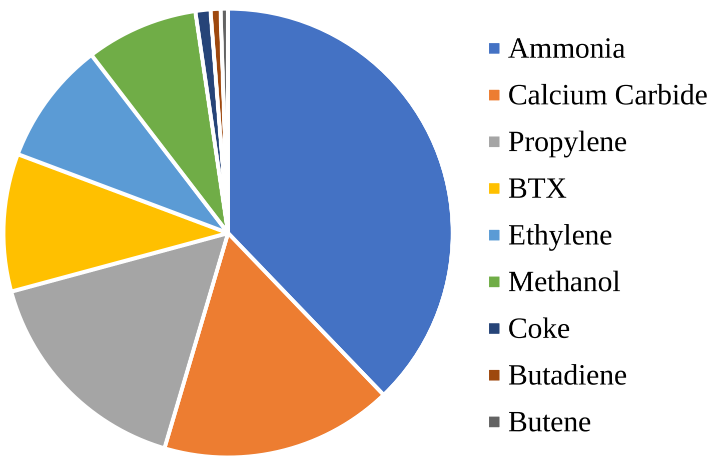

Figure S9. Cumulative carbon emissions breakdown by product

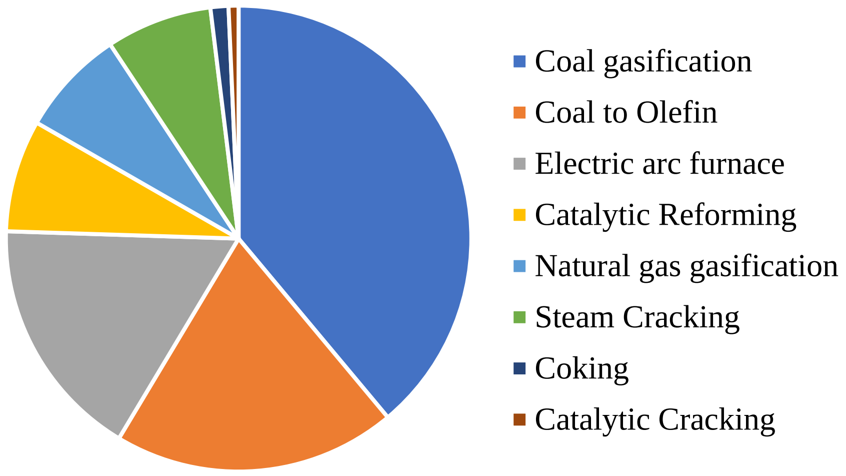

Figure S10. Cumulative carbon emissions breakdown by process

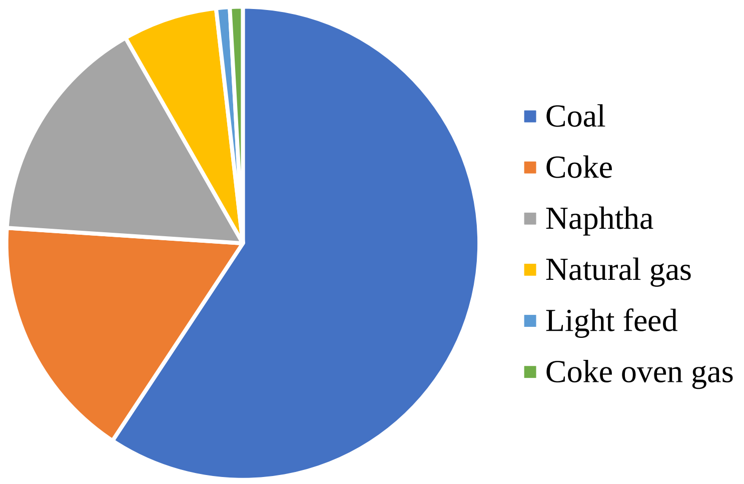

Figure S11. Cumulative carbon emissions breakdown by feedstock

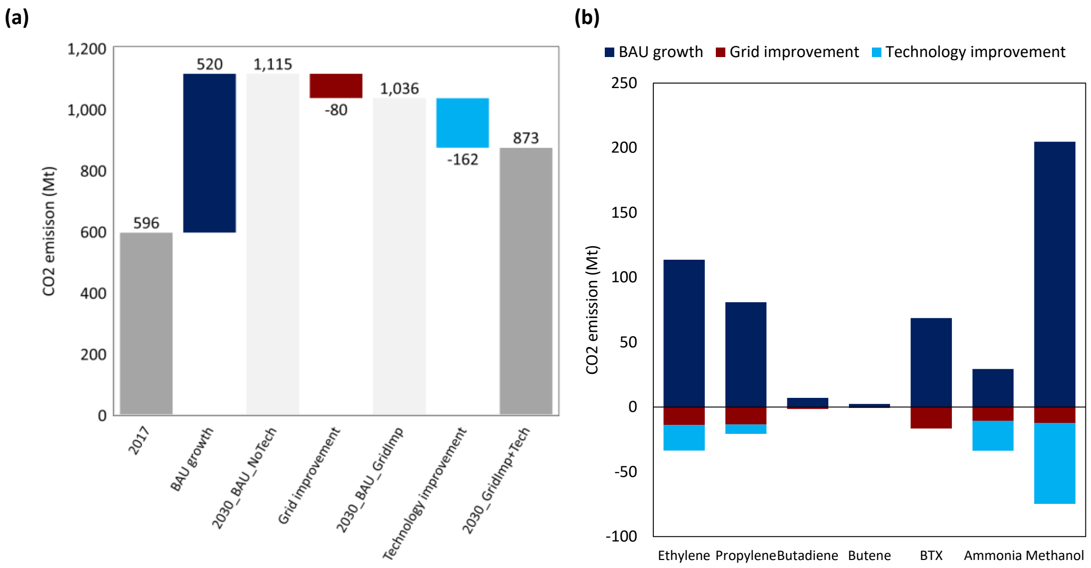

Figure S12. Scenario Analysis up to 2030. (a) Three scenarios for 2030 based on changes since 2017. (b) Categories of emission increases and reductions are classified according to primary chemicals.

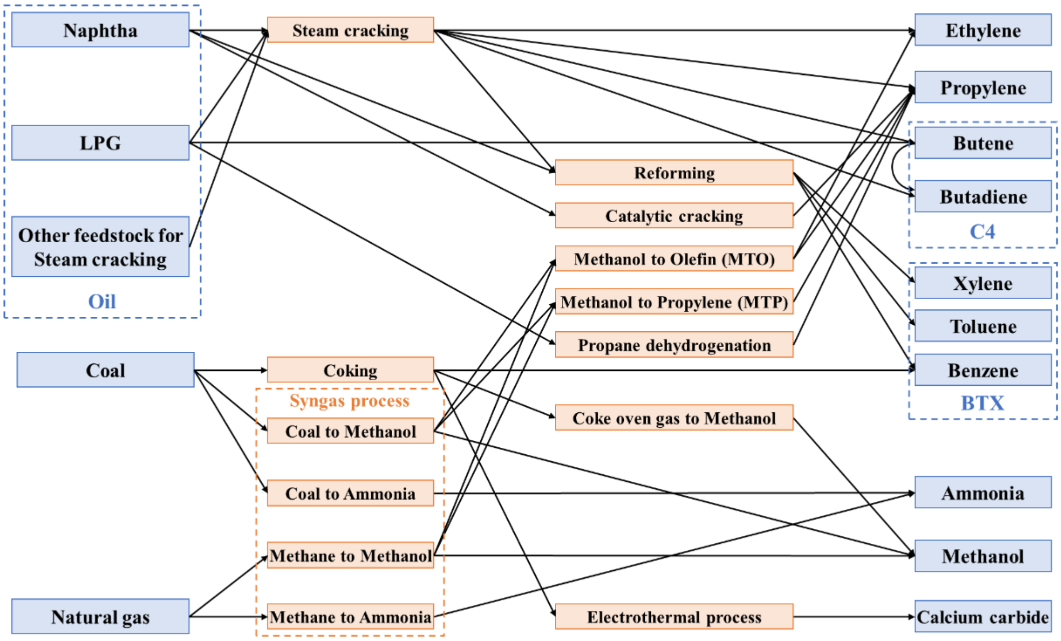

Figure S13. Upstream Material Transformation

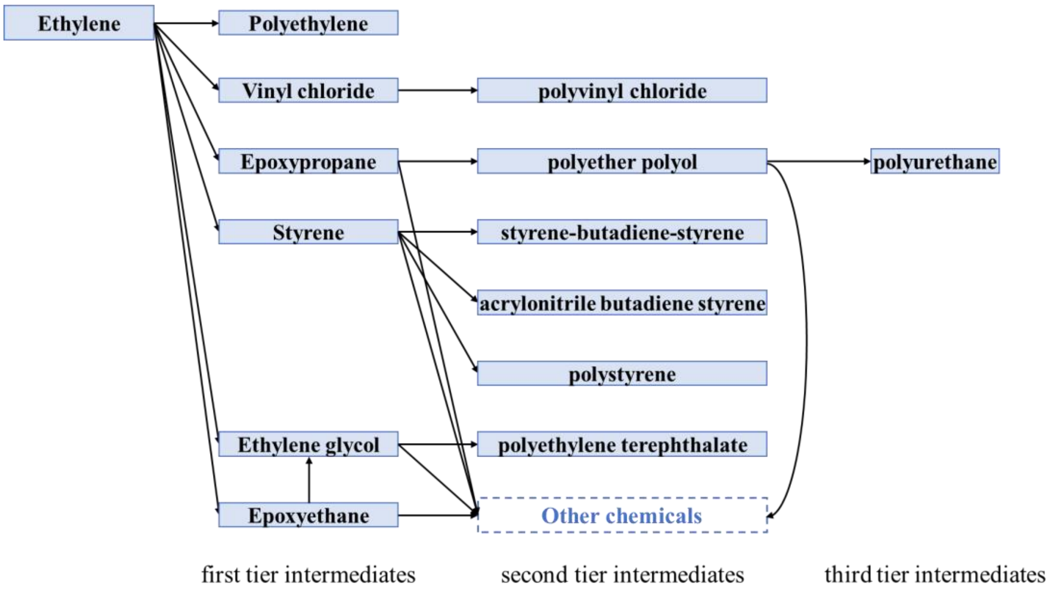

Figure S14. Downstream Material Transformation: Ethylene

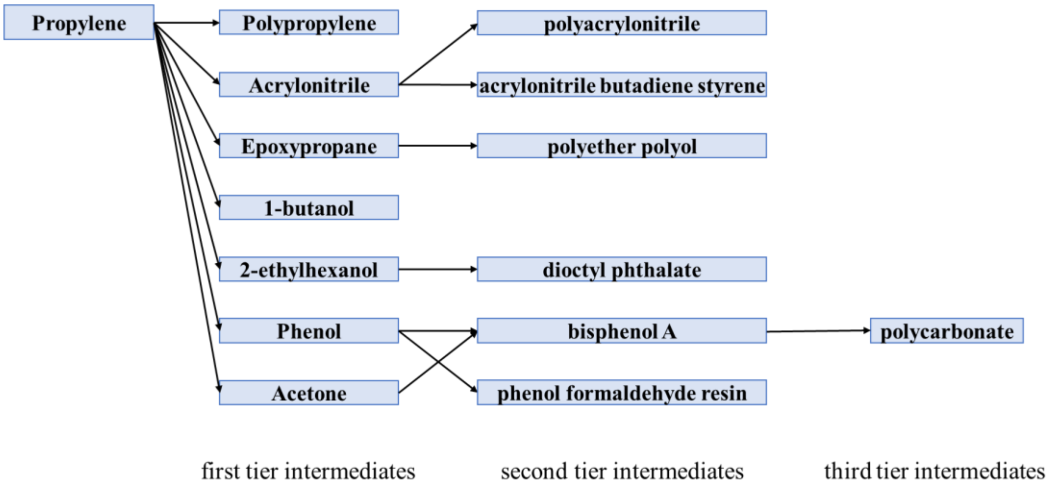

Figure S15. Downstream Material Transformation: Propylene

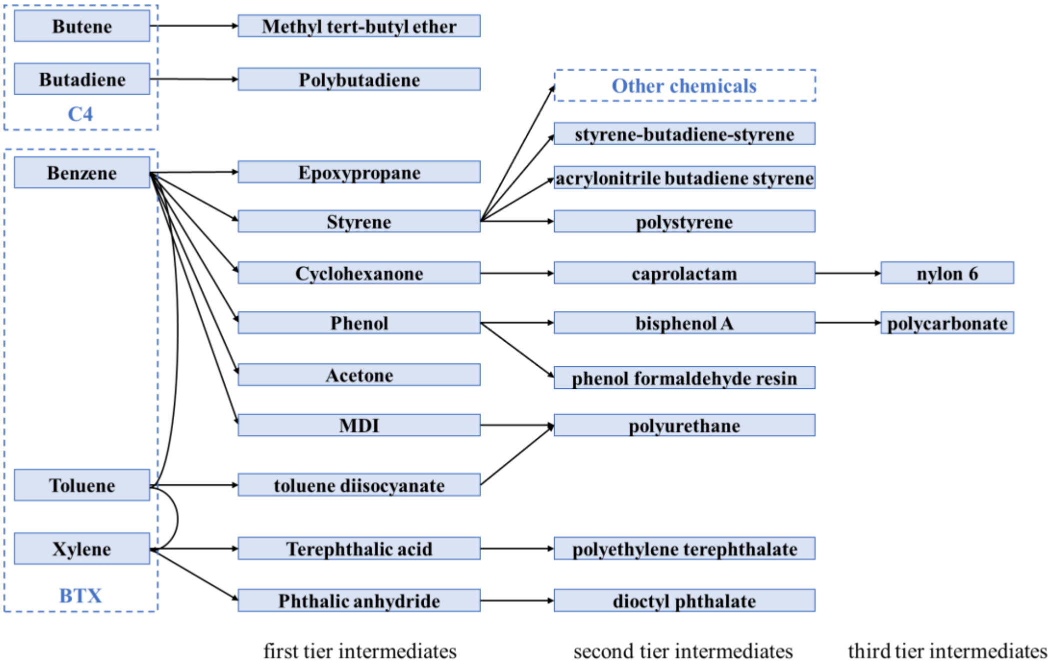

Figure S16. Downstream Material Transformation: C4 and BTX

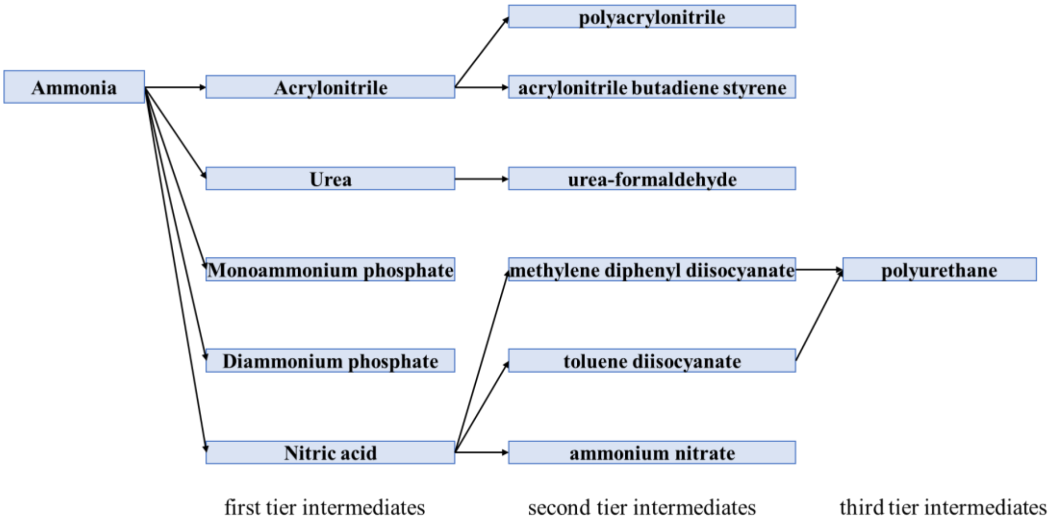

Figure S17. Downstream Material Transformation: Ammonia

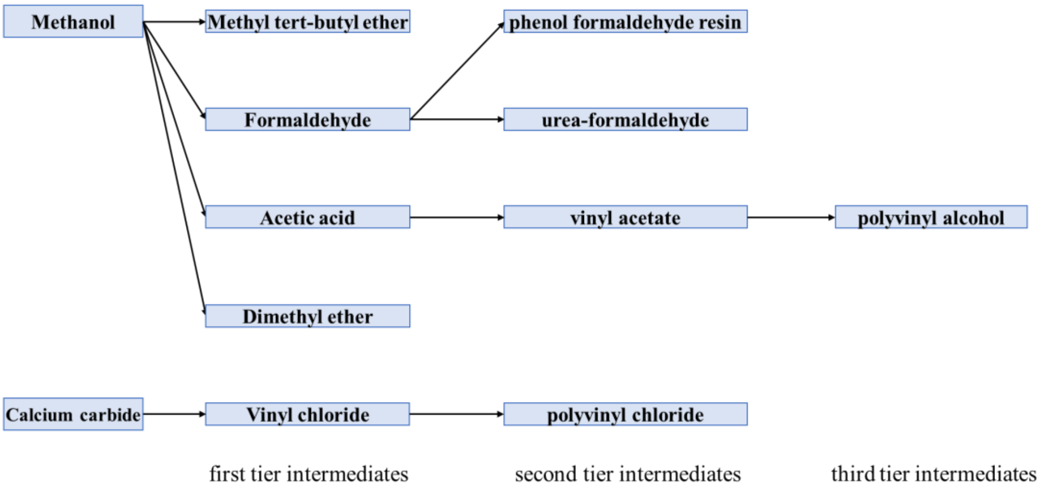

Figure S18. Downstream Material Transformation: Methanol and Calcium Carbide

2. Supplementary Notes

In this section, we outline the methodology for constructing a material flow model to analyze fossil hydrocarbon metabolism. The specific coefficients, processes, and data sources employed are comprehensively detailed in the *Supplementary Data_S2_Flows.xlsx* file.

Acknowledging significant regional variations in the technical parameters of chemical processes and plants within China, our research encompassed an extensive review of technical papers, engineering manuals, factory case studies, and environmental impact assessments, predominantly available in Chinese.

To facilitate accessibility, our reference sheet in *Supplementary Data_S2_Flows.xlsx* includes the original Chinese citations, their English translations, and URLs for reference. The parameters and sources we utilized are systematically documented in various sections of the *Supplementary Data_S2_Flows.xlsx* file.

2.1 Modelling Chemical Flows

Primary chemicals, including ethylene, propylene, C4 olefins (butene, butadiene), BTX (benzene, toluene, o-xylene, m-xylene, p-xylene), methanol, ammonia, and calcium carbide, are the focus of our study. Data on their yield, imports, and exports, along with relevant references, are presented in Table S1.1.1, located in sheet 1.1 of *Supplementary Data_S2_Flows.xlsx*.

We have identified 21 primary chemical production processes and five processes that directly utilize fossil hydrocarbons. These are categorized into three groups, each of which will be discussed in detail in subsequent sections. For comprehensive understanding, the input data and calculation methodology are available in sheets 1.1 to 1.4 of *Supplementary Data_S2_Flows.xlsx*.

2.1.1 Steam cracking and other oil-based processes

<u>(1) Steam cracking</u>

Process description:

- Input - light feedstock (ethane, light diesel, etc.) and heavy feedstock (naphtha, heavy oil, etc.)
- Output - ethylene, propylene, C4 mixture, C6-C8 mixture, others.

Calculation steps:

(1) Calculate the mass of ethylene produced by steam cracking.
(2) Calculate the mass of input and output based on process-based coefficients.
(3) Calculate the indirect carbon emission of steam cracking.

Calculation details:

The computation of chemical processes in this study involves a series of steps utilizing

various data sources. To illustrate the calculation intricacies, we provide an example for each category: oil-based, syngas, and coke-related processes. The complete results for all processes are detailed in Supplementary Data_S2_Flows.xlsx. Note the absolute values presented are in 10kt (10,000 tons) units.

(1) Calculate the mass of ethylene produced by steam cracking:
Ethylene yield from steam cracking (1457.4 [10kt]) = Total ethylene production (1821.8) × the percentage of ethylene from steam cracking (80%).

(2) Calculate the mass of input and output based on process-based coefficients:
i). Total input and output (4364.8) = Ethylene yield from steam cracking (1475.4) ÷ the Proportion of ethylene from steam cracking (33.4%).
ii). Inputs:
Light feed input (593.6) = Total input and output (4364.8) × Proportion of light feedstocks in steam cracking feedstocks (13.6%).
Heavy feed input (3771.2) = Total input and output (4364.8) × Proportion of heavy feedstocks in steam cracking feedstocks (86.4%).
iii). Outputs:
Propylene yield from steam cracking (763.5) = Total input and output (4364.8) × Proportion of propylene in the output (17.5%)
The yields of the other products are calculated similarly for propylene.

(3) Calculate the indirect carbon emission of steam cracking: The calculation process can be found in the “carbon emission” part below.

Note: In actual production, the C4 and C6-C8 mixtures will further undergo an extraction process, resulting in chemicals and residual oil, which may be returned to the cracker as feedstock. In this study, to simplify the calculation, we assumed that the C4 mixtures were all converted to butene and butadiene, and the C6-C8 mixtures were all converted to BTX aromatics.

(2) Catalytic cracking

Process description:

- Input - naphtha.
- Output - propylene.

Calculation steps:

(1) Calculate the mass of propylene produced by catalytic cracking.
(2) Calculate the mass of total input based on process-based coefficients.
(3) Calculate the indirect carbon emission of catalytic cracking.

Note: In the actual production, the catalytic cracking process produces complex products such as gasoline, diesel, etc. and propylene is usually not the main product. In this study, we focused on the feedstock use of fossil hydrocarbons. Therefore, from a mass allocation point of view, we assumed the catalytic cracker supplies propylene only.

(3) Reforming

Process description:

- Input - naphtha.
- Output - BTX aromatics.

Calculation steps:

(1) The naphtha used for steam cracking and catalytic cracking is included in the previous part.
(2) The rest of the naphtha (including import) is all used for reforming.
(3) The mass of BTX aromatics produced by steam cracking is included in the previous part.
(4) Calculate the mass of BTX aromatics produced by reforming.
(5) Calculate the mass of other output based on process-based coefficients.
(6) Calculate the indirect carbon emission of reforming.

(4) Dehydrogenation of propane and butene

Process description:

$$C_3H_8 \rightarrow C_3H_6 + H_2$$

$$C_4H_8 + 0.5\ O_2 \rightarrow C_4H_6 + H_2O$$

Calculation steps:

(1) Calculate the mass of propylene and butadiene produced by dehydrogenation.
(2) Calculate the mass of propane and butene based on process-based coefficients.
(3) Calculate the mass of other input and output based on stoichiometry.
(4) Calculate the indirect carbon emission of dehydrogenation.

2.1.2 Syngas process and methanol-to-olefin

In this study, we expressed coal as $CH_{0.723}$ with a purity of 72.3% [1]; natural gas was written as $CH_{3.886}$ with a purity of 94.4% [2]; and coke oven gas was assumed as CH_6 (CH_4: H_2 = 1:1) with a purity of 95.0%[3]. We use uncertainty (Monte Carlo) simulation to estimate the uncertainty interval brought by different feedstock varieties. Note the absolute values presented are in 10kt (10,000 tons) units.

(1) Ammonia

Process description:

$$6CH_{0.723} + 6H_2O + 3O_2 + 2.723N_2 \rightarrow 5.446NH_3 + 6CO_2$$

$$6CH_{3.886} + 12H_2O + 7.886N_2 \rightarrow 15.772NH_3 + 6CO_2$$

Calculation steps:

(1) Calculate the mass of ammonia produced by the coal-based and gas-based

processes.
(2) Calculate the mass of coal and natural gas based on process-based coefficients.
(3) Calculate the mass of other input and output based on stoichiometry.
(4) Calculate the indirect carbon emission of ammonia production.

Calculation details:

(1) Calculate the mass of ammonia produced by the coal-based and gas-based processes:
   i. The mass of ammonia produced by the coal-based process (4278.6) = Total ammonia production (5629.8) × Proportion of ammonia from the coal-based process (76%).
   ii. The mass of ammonia produced by the gas-based process (1351.2) = Total ammonia production (5629.8) × Proportion of ammonia from the gas-based process (24%).
(2) Calculate the mass of coal and natural gas based on process-based coefficients:
   i. The mass of coal input (5348.3) = The mass of ammonia produced by the coal-based process (4278.6) × Coefficients for the coal-based process to produce ammonia (1.250).
   ii. The mass of natural gas input (878.2) = The mass of ammonia produced by the gas-based process (1351.2) × Coefficients for the gas-based process to produce ammonia (0.650).
(3) Calculate the mass of other input and output based on stoichiometry:

   For example, the mass of nitrogen input in the coal-based process (3856.9) = The mass of coal input (5348.3) × Purity of coal (72.3%) ÷ 12.74 ÷ 6 × 2.732 × 28.0
(4) Calculate the indirect carbon emission of ammonia production.

(2) Methanol

Process description:

$$6CH_{0.723} + 3O_2 + 3.277H_2O \rightarrow 2.723CH_4O + 3.277CO_2$$

$$7.886CH_{3.886} + 6H_2O + 3.772O_2 \rightarrow 7.886CH_4O + 5.658H_2O$$

$$CH_4 + 2H_2 + 1.5O_2 \rightarrow CH_4O + 2H_2O$$

Calculation steps:

(1) Calculate the mass of methanol produced by coal-based, natural gas-based, and coke oven gas-based processes.
(2) Calculate the mass of coal, natural gas, and coke oven gas based on process-based coefficients.
(3) Calculate the mass of other input and output based on stoichiometry.
(4) Calculate the indirect carbon emission of methanol production.

(3) Ethylene glycol (coal-based)

Process description:

$$24CH_{0.723} + 14.723O_2 + 13.108H_2O \rightarrow 5.446C_2H_6O_2 + 13.108CO_2 + 5.446H_2O$$

Calculation steps:

(1) Calculate the mass of ethylene glycol produced by coal-based processes.
(2) Calculate the mass of coal based on process-based coefficients.
(3) Calculate the mass of other input and output based on stoichiometry.

Note: In the actual production, there are four steps in ethylene glycol production [4], including (i)syngas production; (ii)methyl nitrite production; (iii)dimethyl oxalate production; (iv)dimethyl oxalate reduction. Here we gave a simplified overall reaction to the four steps.

(4) Hydrogen, Carbon monoxide, and Synthetic gas (coal-based)

Process description:

$$2CH_{0.723} + 2H_2O + O_2 \rightarrow 2.723H_2 + 2CO_2$$

$$4CH_{0.723} + 2.723O_2 \rightarrow 4CO + 1.446H_2O$$

$$6CH_{0.723} + 3O_2 + 3.277H_2O \rightarrow 2.723\ (CO + 2H_2) + 3.277CO_2$$

Calculation steps:

(1) Calculate the mass of hydrogen, carbon monoxide, and synthetic gas (CO: H_2 = 1:2) produced by coal-based processes.
(2) Calculate the mass of input and output based on stoichiometry.

(5) Methanol-to-Olefin

Process description:

$$2CH_4O \rightarrow C_2H_4 + 2H_2O$$

$$3CH_4O \rightarrow C_3H_6 + 3H_2O$$

Calculation steps:

(1) Calculate the mass of ethylene produced by the MTO process.
(2) Calculate the mass of propylene produced by the MTO and MTP processes.
(3) Calculate the mass of methanol based on process-based coefficients.
(4) Calculate the mass of other input and output based on stoichiometry.
(5) Calculate the indirect carbon emission of coal-to-olefin processes.

Note: In actual production, there are two methanol-to-olefin processes: MTO (product: ethylene: propylene = 1:1) and MTP (product: nearly pure propylene)[5]. In this study, from the perspective of mass allocation, we assume MTO as a combination of both reactions and MTP as reaction 2.

2.1.3 Coke, calcium carbide, and vinyl acetate production

(1) Calcium carbide (Electrothermal process)

Process description:

$$CaCO_3 + 3C + 0.5O_2 \rightarrow CaC_2 + 2CO_2$$

Calculation steps:

(1) Calcium carbide is all produced by the electrothermal process.
(2) Calculate the mass of coke and calcium carbonate based on process-based coefficients.
(3) Calculate the mass of other input and output based on stoichiometry.
(4) Calculate the indirect carbon emission of electrothermal processes.

Calculation details:

(1) Calcium carbide is all produced by the electrothermal process, i.e. The mass of carbide produced by the electrothermal process (2673.0) = Total carbide production (2673.0).
(2) Calculate the mass of coke and calcium carbonate based on process-based coefficients:
    i. The mass of coke input (1603.8) = The mass of carbide produced by the electrothermal process (2673.0) × Coefficients for the electrothermal process to produce carbide (0.600).
    ii. The mass of calcium carbonate input (5346.0) = The mass of carbide produced by the electrothermal process (2673.0) × Coefficients for the electrothermal process to produce carbide (2.000).
(3) Calculate the mass of other input and output based on stoichiometry:
(4) For example, the mass of oxygen input in the electrothermal process (663.8) = The mass of coke input (1603.8) × Purity of coke (93.2%) ÷ 12.01 ÷ 3 × 0.5 × 32.0
(5) Calculate the indirect carbon emission of electrothermal processes.

Note: In actual production, there are three steps in calcium carbide production, including (i)thermal decomposition of limestone; (ii)formation of calcium carbide; and (iii)disposal of carbon monoxide. Here we present the overall reaction of the three steps.

(2) Coke (Coking)

Process description: Input - coal. Output - coke, benzene, coke oven gas.

Calculation steps:

(1) The mass of coke, benzene (coal-based), and coke oven gas is calculated in the previous process.
(2) Calculate the mass of total input based on process-based coefficients.
(3) Calculate the indirect carbon emission of coking.

Note: In actual production, the coking process has a certain proportion of output composition. In this study, we distribute them proportionally according to the mass allocation.

(3) Vinyl acetate (Methane-based process)

Process description:

$$180CH_4 + 121O_2 + 32C_2H_4O_2 \rightarrow 32C_4H_6O_2 + 14CO_2 + 102CO + 216H_2 + 112H_2O$$

Calculation steps:

(1) Calculate the mass of vinyl acetate produced by methane-based processes.
(2) Calculate the input and output based on stoichiometry.

Note: In the actual production, there are two steps in vinyl acetate produced from methane, including (i) thermal decomposition of methane and (ii) addition of acetylene and acetic acid[6]. Besides methane-based processes, vinyl acetate can also be produced by the ethylene-based process and calcium carbide-based process, which is given in the second part of the calculation.

2.2 Trace Production Mix and Feedstock Intensity

Further details about consumption mix (CM):

$CM_{coal,i-1}$: Adjusted production mix considering the production mix of imported and exported chemicals for chemical $i-1$.

Taking benzene as an example, $PM_{coal,benzene} = 26\%$. According to experts' suggestions, the benzene imported into China was oil-based. Consequently, after the adjustment, $CM_{coal,benzene} = 21\%$.

Moreover, the products of a particular process may have only an exclusive use due to the design of the factory (especially for Refinery-Chemical Integration). For example, ethylene and propylene produced from coal are generally only used for polyethylene and polypropylene production typically. Those coal-based ethylene and propylene are not used for epoxyethane or acrylonitrile production.

The PM is given in ***Figure 3*** and ***Figure 4***. See ***Figure S5 - S7*** for the CM which includes the import/export.

The FI (feedstock intensity) of eight synthetic polymers, the yield of recycled plastic pellets, and theoretical fossil feedstock conservation are presented in *Supplementary Data_S2_Flows.xlsx*.

2.3 Carbon Emissions of Primary Chemicals Production

In this study, we apply the coefficient-based carbon accounting method to check the scope 2 carbon emissions for the 21 primary chemical production processes. The process-based carbon emission has been calculated using stoichiometry. For the energy-based carbon emission, use the following equation:

$$CO_{2,ind} = \sum_{j=e,h} Q_j \cdot E_j$$

The electricity emission factor Q_e: 4.880 tCO_2/tce. It is calculated as follows:

We used the average emission factor of the power grid as published by the Chinese government (*Climate Change of the People's Republic of China Second Biennial Update Report (2018)*): https://www.mee.gov.cn/ywgz/ydqhbh/wsqtkz/201907/P020190701765971866571.pdf; and *Guidelines for Corporate Greenhouse Gas Emissions Accounting Methodology and Reporting - Power Generation Facilities (2021 version)*: https://www.mee.gov.cn/xxgk2018/xxgk/xxgk06/202112/W020211202787049808223.pdf). For reference, this factor was 0.6101 tCO2/MWh in 2015 and reduced to 0.5839 tCO2/MWh by 2020. In this way, we can derive the compound annual growth rate (CAGR) of the emission factor as:

$$CAGR = \sqrt[(2020-2015)]{\frac{0.5839}{0.6101}} - 1 = -0.874\%$$

So the 2017 carbon emission factor can be calculated as:

$$0.6101 \times (1 - 0.874\%)^2 = 0.5995 \ \mathrm{tCO_2/MWh}$$

In this study, due to the non-uniform original data units and confidentiality of some data, we opted to use tons of standard coal (tce) as the consistent unit for our emission coefficients. The "ton of standard coal" is a unit frequently employed in Chinese industrial production, symbolizing any energy source with a calorific value of 7×10^6 kcal. Thus, the conversions are:

$$1 \ \mathrm{tce} = 7 \times 10^6 \mathrm{kcal} = 29307.6 \ \mathrm{MJ} = 8141 \ \mathrm{kWh}$$

Transforming this emission factor into the unit of tCO2/tce, we obtain the emission coefficient grounded on tce as:

$$Q_e = 0.5995 \mathrm{tCO_2/MWh} \times 8.141 \mathrm{MWh/tce} = 4.880 \ \mathrm{t\,CO_2/tce}$$

The heat emission factor Q_h: 2.870 tCO_2/tce (Calculated according to the energy mix of heat supply)[7].

The electricity consumption E_e (tce/t) and heat consumption E_h (tce/t): from plant data. The units of raw data include MJ, kWh, tce, etc. Some plant data is confidential. The harmonized energy consumption data (tce/t) are presented in Table S1.1.2 in

sheet 1.1 of *Supplementary Data_S2_Flows.xlsx*.

Here are two examples for the coefficient-based carbon accounting:

2.3.1 Coal-based ammonia production

Coal-based ammonia production is a process characterized by significant direct emissions. This example serves to demonstrate the calculation of carbon emissions for a single process. A schematic diagram is used to illustrate the various stages of this process:

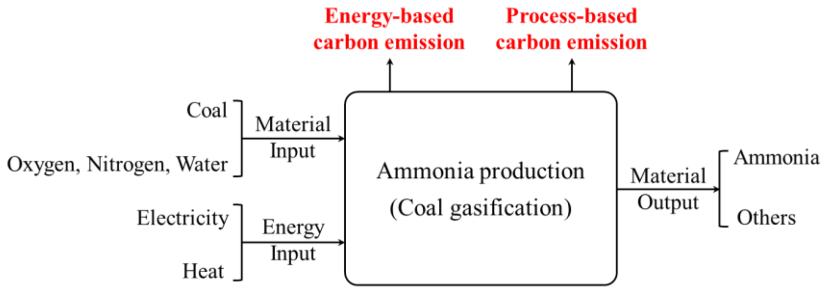

The following mass balance result is retrieved from sheet 1.3 in Supplementary Data S2:

Input	Mass (10kt)	Output	Mass (10kt)
Coal	5348.3	NH_3	4278.6
H_2O	5463.3	CO_2	13354.8
O_2	3237.5	loss	272.6
N_2	3856.9	**sum**	17906

So the direct carbon intensity can be calculated as follows:

$$CO_{2,dir} = \frac{total\ emissions}{product\ quantity} = \frac{13354.8\ \mathrm{10ktCO_2}}{4278.6\ \mathrm{10ktNH_3}} = 3.12\ \mathrm{tCO_2/tNH_3}$$

The total electricity and heat consumption to produce 1t NH_3 (from the plant data) is:

Electricity consumption: 0.184 tce

Heat consumption: 0.159 tce

We can calculate the E_e and E_h to produce 1t NH_3 as follows:

$$E_e = \frac{total\ electricity\ consumption}{product\ quantity} = \frac{0.184\ \mathrm{tce}}{\mathrm{1tNH_3}} = 0.184\ \mathrm{tce/tNH_3}$$

$$E_h = \frac{total\ heat\ consumption}{product\ quantity} = \frac{0.159\ \mathrm{tce}}{\mathrm{1tNH_3}} = 0.159\ \mathrm{tce/tNH_3}$$

The indirect carbon intensity can be calculated as follows:

$$CO_{2,ind} = \sum_{j=e,h} Q_j \cdot E_j$$
$$= 4.88 \mathrm{tCO_2/tce} \times 0.184 \mathrm{tce/tNH_3} + 2.87 \mathrm{tCO_2/tce} \times 0.159 \mathrm{tce/tNH_3}$$
$$= 1.36\ \mathrm{tCO_2/tNH_3}$$

The total carbon intensity of the coal-to-ammonia process can be calculated as follows:

$$CO_2 = CO_{2,dir} + CO_{2,ind} = 3.12\ \mathrm{tCO_2/tNH_3} + 1.36\ \mathrm{tCO_2/tNH_3} = 4.48\ \mathrm{tCO_2/tNH_3}$$

The total carbon emission of the coal-to-ammonia process can be calculated as follows:

$$CO_{2,total} = CO_2 \times product\ quantity = 4.48\ \mathrm{tCO_2/tNH_3} \times 4278.6\ \mathrm{10ktNH_3}$$
$$= 192\ \mathrm{MtCO_2}$$

We can also calculate the total indirect carbon emission:

$$CO_{2,ind,total} = CO_{2,ind} \times product\ quantity = 1.36\ \mathrm{tCO_2/tNH_3} \times 4278.6\ \mathrm{10ktNH_3}$$
$$= 58\ \mathrm{MtCO_2}$$

2.3.2 Steam cracking

Steam cracking, a process with negligible direct emissions and multiple products, serves as an example to illustrate our allocation method. This method mirrors that used in refinery processes, where the Share of Mass Content (SMC) of each product is calculated for allocation[8]. A schematic diagram of the process is presented to detail this approach:

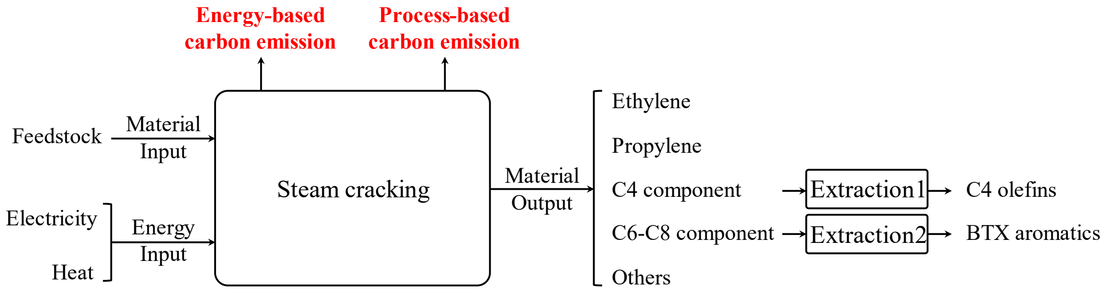

The following mass balance result for the steam cracking unit is retrieved from sheet 1.2 in Supplementary Data S2:

Input	Mass (10kt)	Output	Mass (10kt)

Light feed	593.6	Ethylene	1457.4
Heavy feed	3771.2	Propylene	763.5
		C4	411.9
		C6-C8	770.5
**sum**	4364.8	others	961.5

Here we assume the direct carbon intensity is 0 for steam cracking.

The total electricity and heat consumption for the steam cracking unit to produce 1t ethylene (average of the light feed and heavy feed, from the plant data) is:

Electricity consumption: 0.051 tce; Heat consumption: 1.068 tce

The total electricity and heat consumption for the extraction1 unit to produce 1t C4 olefins from 1t C4 components (from the plant data) is:

Electricity consumption: 0.016 tce; Heat consumption: 0.165 tce

The total electricity and heat consumption for the steam cracking unit to produce 1t BTX aromatics from 1t C4-C8 component (from the plant data) is:

Electricity consumption: 0.011 tce; Heat consumption: 0.146 tce

We set the quantity of ethylene to 1 and we can get the normalized table:

Input	Mass (t)	Output	Mass (t)
Light feed	0.4	Ethylene	1.0
Heavy feed	2.6	Propylene	0.5
		C4	0.3
		C6-C8	0.5
**sum**	3.0	others	0.7

We allocate the carbon emission of the process to all the products (ethylene, propylene, C4, C6-C8, and others). According to the principle of mass allocation, the energy consumption intensity $E_{e,h}$ (tce/t) of each product (output) in the steam cracking unit is equivalent. We can calculate them as follows:

$$E_{e,cracking} = \frac{total\ electricity\ consumption}{product\ quantity}$$
$$= \frac{0.051\ \text{tce}}{(1 + 0.5 + 0.3 + 0.5 + 0.7)\ \text{t product}} = 0.017\ \text{tce/t product}$$
$$E_{h,cracking} = \frac{total\ heat\ consumption}{product\ quantity} = \frac{1.068\ \text{tce}}{(1 + 0.5 + 0.3 + 0.5 + 0.7)\ \text{t product}}$$
$$= 0.356\ \text{tce/t product}$$

The energy intensity for the extraction unit can be calculated as follows

$$E_{e,extraction1} = \frac{total\ electricity\ consumption}{product\ quantity} = \frac{0.016\ \text{tce}}{1\ \text{t product}}$$
$$= 0.016\ \text{tce/t product}$$
$$E_{h,extraction1} = \frac{total\ heat\ consumption}{product\ quantity} = \frac{0.165\ \text{tce}}{1\ \text{t product}} = 0.165\ \text{tce/t product}$$
$$E_{e,extraction2} = \frac{total\ electricity\ consumption}{product\ quantity} = \frac{0.011\ \text{tce}}{1\ \text{t product}}$$
$$= 0.011\text{tce/t product}$$
$$E_{h,extraction2} = \frac{total\ heat\ consumption}{product\ quantity} = \frac{0.146\ \text{tce}}{1\ \text{t product}} = 0.146\ \text{tce/t product}$$

The carbon intensity and carbon emission of each product can be then calculated:

Ethylene:

$$CO_{2,ind,ethylene} = \sum_{j=e,h} Q_j \cdot E_j$$
$$= 0.017\ \text{tce/t} \times 4.880\ \text{tCO}_2\text{/tce} + 0.356\ \text{tce/t} \times 2.870\ \text{tCO}_2\text{/tce}$$
$$= 1.10\ \text{tCO}_2\text{/t C}_2\text{H}_4$$
$$CO_{2,ethylene} = CO_{2,ind,ethylene} \times product\ quantity$$
$$= 1.10\ \text{tCO}_2\text{/tC}_2\text{H}_4 \times 1457.4\ \text{10ktC}_2\text{H}_4 = 16\ \text{MtCO}_2$$

Propylene:

$$CO_{2,ind,propylene} = CO_{2,ind,ethylene} == 1.10\ \text{tCO}_2\text{/t C}_3\text{H}_6$$
$$CO_{2,propylene} = CO_{2,ind,propylene} \times product\ quantity$$
$$= 1.10\ \text{tCO}_2\text{/tC}_3\text{H}_6 \times 763.5\ \text{10ktC}_3\text{H}_6 = 8.4\text{MtCO}_2$$

C4 olefin:

$$CO_{2,ind,C4\ olefin} = CO_{2,ind,ethylene} + \sum_{j=e,h} Q_{j,extraction1} \cdot E_{j,extraction1}$$
$$= 1.10\ \text{tCO}_2\text{/t C4} + 0.016\ \text{tce/t} \times 4.880\ \text{tCO}_2\text{/tce}$$
$$+ 0.165\ \text{tce/t} \times 2.870\ \text{tCO}_2\text{/tce} = 1.65\ \text{tCO}_2\text{/t C4}$$

$$CO_{2,C4\ olefin} = CO_{2,ind,C4\ olefin} \times product\ quantity$$
$$= 1.65\ \mathrm{tCO_2/tC4} \times 411.9\ \mathrm{10kt\ C4} = 6.8\ \mathrm{MtCO_2}$$

BTX aromatics:

$$CO_{2,ind,BTX\ aromatics} = CO_{2,ind,ethylene} + \sum_{j=e,h} Q_{j,extraction2} \cdot E_{j,extraction2}$$
$$= 1.10\ \mathrm{tCO_2/t\ C4} + 0.011\ \mathrm{tce/t} \times 4.880\ \mathrm{tCO_2/tce}$$
$$+ 0.146\ \mathrm{tce/t} \times 2.870\ \mathrm{tCO_2/tce} = 1.58\ \mathrm{tCO_2/t\ BTX}$$

$$CO_{2,BTX\ aromatics} = CO_{2,ind,BTX\ aromatics} \times product\ quantity$$
$$= 1.55 \frac{\mathrm{tCO_2}}{\mathrm{tBTX}} \times 770.5\ \mathrm{10kt\ BTX} = 1.2\ \mathrm{MtCO_2}$$

2.4 Uncertainty Analysis

Diverse refineries and chemical plants, even within the same vicinity, often operate under differing process parameters. We employed an uncertainty analysis to gauge the potential deviation in results, using Monte Carlo simulations for uncertainties in our material flow model [9,10].

In our MFA modeling process, there are mainly two types of data inputs:

- Product Outputs (Yield Data): Assumed to be accurate as sourced from the official yearbook.
- Process-Based Coefficient Data: Including product-to-feedstock ratios, which were our primary focus due to their uncertainties.

We employed normally distributed independent random variables to describe all uncertain coefficients and parameters, basing the baseline value on our initial guess. This value was set as the mean of the normal distribution. The uncertainty range was ascertained from quality sources, factory data, literature, and expert opinions (see Supplementary Notes and Supplementary Datasets S2 for details). We used 20,000 Monte Carlo simulations to deduce a final confidence value, choosing the 95% certainty level for results. In a word, we used [1/6*deviation range] (for example, 0.4/6 for a deviation range of ±20%) as the standard deviation in this approach to cover 99.7% of the range of change (within 3 standard deviations in a normal distribution). Specific data from Statistical Yearbooks and results with a deviation of less than 0.5% were excluded from the uncertainty range.

For primary chemicals production—primarily the conversion of hydrocarbons from fossil fuels—uncertainty was higher. National standards provided values for these processes, categorizing them into "threshold", "standard", and "advanced" energy/fossil resource consumption levels. Each level denotes a different efficiency tier in production:

- Threshold value: Minimum efficiency for new production ventures. The level that new production capacity, such as new construction, renovation, expansion projects, etc., must meet. Its value should, in theory, represent the last 20% of the industry's energy efficiency level.
- Standard value: The required efficiency level for existing businesses. The value should be based on the elimination of a certain percentage of existing high-energy-consuming backward production capacity. Approximately 20% of backward products and production capacity should be eliminated as a result of energy-saving transformation.
- Advanced value: It benchmarks the leading energy efficiency level of the same type of production.

Given the mandatory nature of these national standards, we consider them highly representative of reality. Chemical reactions in downstream production, being well-defined, present lower uncertainty.

The uncertainty range data are given in four ways:

(1) For primary chemicals production and processes directly consuming fossil hydrocarbons, we use data from the *National Standard of the People's Republic of China* (see Supplemental Table S3-S10 below). The series of standards *Norm of energy consumption per unit product* gives the Threshold value, Standard value, and advanced value of energy consumption per unit product. We use the larger range of Threshold value and Advanced value from the Standard value. If there is no standard for a process, use 5% for the uncertainty range.
(2) For PE, PP, PVC, PS, and butadiene rubber production, the material consumption is close to the thermodynamic limit. Therefore, we consider the uncertainty range as 1%.
(3) For fertilizer and coal-based vinyl chloride production, different formulations can bring about large changes in raw material inputs. Therefore, we consider the uncertainty range as 10%.
(4) For other processes, a general uncertainty range of 5% is assumed.

For a detailed uncertainty range of each point, refer to Supplementary Data_S2_Flows.xlsx.

2.5 Quantify Future Emission Reduction Potential to 2030

Here, we construct a framework designed to estimate emissions associated with a range of platform compounds in China by the year 2030. Key features of this framework include:

- Projections of production volumes for 2030
- Assessment of the proportion of process routes, incorporating emerging technologies (routes share)
- Enhancements in energy efficiency, inclusive of grid decarbonization (coefficients)
- Advancements in process technology (coefficients)

We delineated three distinct scenarios for comparison: (1) Baseline Scenario: No change in intensity factors or technology; (2) Grid Improvement Scenario: Incorporating grid purification and energy mix improvements; (3) Technology Scenario: Introduction of technologies such as green hydrogen, CCUS and shift in feedstock mix. Below we provide a brief introduction to the methods and data sources. Detailed results and parameters are included in *Supplementary Data_S2_Flows.xlsx.*

2.5.1 Grid Decarbonization (Coefficient)

Here we used the similar CAGR method to estimate the average emission factor for China's National Grid in 2030 based on the average emission factor in 2020 and 2022.

The 2020 average emission factor is from the *Guidelines for Corporate Greenhouse Gas Emissions Accounting Methodology and Reporting - Power Generation Facilities (2021 version)*, which is 0.5839 tCO2/MWh: https://www.mee.gov.cn/xxgk2018/xxgk/xxgk06/202112/W020211202787049808223.pdf).

The 2022 average emission factor is from the *Notice on the Work Related to the Management of Greenhouse Gas Emission Reporting for Enterprises in the Power Generation Sector in 2023-2025*, which is 0.5703 tCO_2/MWh: https://www.mee.gov.cn/xxgk2018/xxgk/xxgk06/202302/t20230207_1015569.html

The CAGR can be calculated as follows:

$$CAGR = \sqrt[(2022-2020)]{\frac{0.5703}{0.5839}} - 1 = -1.171\%$$

The 2030 carbon emission factor can be calculated as:

$$0.5703 \times (1 - 1.171\%)^8 = 0.5190 \ \mathrm{tCO_2/MWh}$$

Transforming this emission factor into the unit of tCO2/tce, we obtain the emission coefficient grounded on tce as:

$$Q_e = 0.5190 \ \mathrm{tCO_2/MWh} \times 8.141 \mathrm{MWh/tce} = 4.225 \ \mathrm{t\,CO_2/tce}$$

2.5.2 Process and Other Energy Improvements Coefficient

The sources of the emission factors include the China National Petroleum & Chemical Planning Institute (NPCPI)[11-14], McKinsey[15], the Rocky Mountain Institute (RMI)[16], and literature[17-24].

2.5.3 Production Volumes & Routes Share

(1) Ethylene

RMI[16] projects that by 2030, China's ethylene production will reach 77 million tons. And, the industry experts we consulted predict a more conservative figure of 73 million tons. According to their assessments, 87% of this production is anticipated to rely on heavy catalytic cracking, which includes both heavy and light feedstocks. Meanwhile, 13% is expected to be produced via MTO/CTO processes. Within this latter category, 1% of olefins are projected to be derived directly from the syngas approach, and an additional 1% from a process that combines green hydrogen with coal-to-ethylene/MTO. Research[17] into China's coal chemical industry indicates that integrating green hydrogen into the coal-to-olefin process can achieve a substantial CO2 emission reduction ratio of 67.9%. According to expert estimates, olefins production directly from syngas can reduce carbon dioxide by 20% compared to direct MTO.

(2) Propylene

The production data for propylene primarily comes from expert estimates, amounting to 72.9 Mt. This figure is principally projected based on the growth rate from 2017 to 2022, taking into account a decrease in the growth rate. The following are the estimated proportions for each production route. Steam Cracking is projected to have the largest share at 41%. Catalytic cracking follows with a 19% share. Propane dehydrogenation is expected to account for 25% of the production. Coal-to-propylene/MTO has a projected share of 9%, while Coal-to-propylene/MTP is estimated at 2%. Olefins production directly from syngas is forecasted to contribute 1%, which is also the case for another variant of olefins production directly from syngas. Lastly, both green hydrogen-coupled coal-to-propylene/MTO and green hydrogen-coupled coal-to-propylene/MTP are each anticipated to make up 1% of the production.

(3) C4

The estimated production of C4 largely relies on the GDP growth rate and expert recommendations. Our projects suggested 6.89 Mt of Butadiene and 3.40 Mt of Butene by 2030. Due to poor economic viability, the proportion of Butene oxidation is approximately 8%. Catalytic cracking of C4 will constitute the majority share at 92%.

(4) BTX

The production data for BTX (Benzene, Toluene, Xylenes) is derived from source [25] and cross-verified by experts, reaching 72.86 Mt. The proportion of coal (coking benzene) is expected to continue declining to 5% by the year 2030. The petroleum route is estimated to account for approximately 95% of the production.

(5) Ammonia

There are different forecasts for ammonia production in 2030. The Rocky Mountain Institute (RMI) [16] estimates production at 48 Mt, and McKinsey[15] estimates production at 50.1 Mt. Based on our discussions with industry experts, it is expected that ammonia production may increase from 2020 to 2025, and there may be a period of slow growth, stability or even decline from 2025 to 2030. However, based on the current new production capacity and demand, Sinopec has raised its forecast for synthetic ammonia production in 2030. Therefore, we use 63 Mt as the predicted value here.

RMI[16] envisions that by 2030, the proportion of coal-based synthetic ammonia will decrease to 70%, with 20% of synthetic ammonia potentially coming from the rapidly developing Power-to-X (PtX) pathways, and over 30% requiring Carbon Capture and Storage (CCS) technology. Experts we consulted recommend that 67% of production could come from traditional synthetic ammonia methods, 10% could be coupled with green hydrogen synthesis and 22% might involve coal gasification combined with ammonia synthesis.

However, there is some uncertainty in these projections, as developments in the integration of new energy sources and clean coal conversion could influence these figures. According to SinoCarbon and Energy Foundation[26], from the present until 2025, there will be a focus on the combined use of new energy and the clean conversion of coal. From 2025 to 2030, the modern coal chemical industry will gradually begin to deeply integrate with renewable energy hydrogen production. Although there are differing opinions in the industry on the proportion of coal-to-chemical plants that can integrate green hydrogen, it is generally accepted as an effective way to significantly reduce coal-to-chemical emissions. Ammonia for energy storage will enter a period of rapid development after 2030[16], accounting for 50% by 2050, and will be the main driving force for the development of the synthetic ammonia industry in the future. In our analysis based on 2030, ammonia for energy storage is not considered.

(6) Methanol

China National Petroleum & Chemical Planning Institute (NPCPI)[11-14] and RMI[16] project the production volumes of methanol in 2030 to be around 50-60 Mt.

RMI[16] posits that during the production increase phase from 2020 to 2030, the

additional output will primarily come from zero-carbon production pathways such as Power-to-X (PtX). However, due to the inertia of planning, a portion of the increment will still be provided by the coal chemical industry, but it will need to be equipped with Carbon Capture and Storage (CCS) or coupled with green hydrogen to minimize carbon emissions to the greatest extent possible. Among all coal-based capacities, 30% to 40% will either be coupled with green hydrogen or equipped with CCS. However Chinese industry experts may think this ratio is too optimistic in 2030. We estimate that only 5% of methanol production capacity will come from green hydrogen. Biomass-to-methanol, due to its limited sustainable supply and higher cost, will only account for less than 1% of the total production and hence not included here. Taking into account various sources, we have set the following proportions for China's methanol production sources by 2030. Coal-to-methanol is expected to be the predominant source at 74%. Carbon dioxide hydrogenation will contribute a small fraction, at 1%. Green hydrogen-coupled coal-to-methanol is projected to account for 5% of the production. Both natural gas to methanol and coke oven gas to methanol are anticipated to each have a 10% share in the production landscape.

(7) Calcium Carbide & Coke

China does not encourage high-carbon routes such as calcium carbide and coking. We have assumed a slow growth rate for these industries, and reach 30 Mt by 2030 for calcium carbide. However, recent technological breakthroughs in the use of mercury-free catalysts in the calcium carbide process of PVC production[27] may increase the output of calcium carbide-based PVC production.

(8) CCUS

CCUS, with a potential for 50 Mt emission reduction and subject to industry debate due to external factors[21], is considered in aggregate rather than by distinct technology.

(9) Other Notes

About biomass-route and chemical recycling route to produce primary chemicals:

While biomass-derived chemicals hold promise for emissions reduction, their contribution might be limited to 1-3% by 2030 in China[22] and less than 10% globally by 2050[28]. Since routes involving biomass and CO_2 catalytic hydrogenation are expected to undergo commercialization or be put into production in actual factories post-2030[22], they are not included in this analysis.

Recycling and a circular economy could diminish the demand for raw materials. Given our framework directly estimates the primary production volumes, it does not include the recycling flows.

High CO_2 concentrations in chemical production make carbon capture a cost-effective option, yet by 2030, CCUS might offset only 30-50 Mt of CO_2 in the chemical domain

in China[21]. Considering methanol production through CO_2 hydrogenation could reshape the low-carbon chemical landscape[29], however, supplying abundant, cost-effective green electricity for hydrogen and CCUS generation remains uncertain[30,31], and larger emission reductions can be attained if this electricity is used to replace coal power plants. Transitioning from fossil hydrocarbons can gain momentum through circular chemical systems[32] and biomass feedstock[28]. With China's recycled plastic pellet output in 2019 standing at 16 Mt, and considering a current recycling efficiency of 30%, this could theoretically displace 12 Mt of coal and crude oil each as primary feedstock[33]. Chemical recycling has the potential, but technology complexities suggest that only 11-17% of the demand might be satisfied by recycled plastics[34]. While biomass-derived chemicals hold promise for emissions reduction, their potential contribution is expected to be only 1-3% by 2030 in China[22] and less than 10% globally by 2050[28], limited by biomass availability and the rate of investment. The high costs present a formidable barrier.

2.6 Other CO_2 Emission Mitigation Potential and Cases of Alternative Technologies

2.6.1 CO_2 Emission Reduction Potential of Primary Chemical Production

We also collect the CO_2 emission reduction potential of primary chemicals production of hydrogen/CO_2, biomass, and CCS that are not included in our 2030 framework due to the technology readiness. Please see *Data_S2_Flows.xlsx* for detailed data.

For analysis and visualization of how the coal chemical industry can be integrated with green hydrogen and green electricity geographically, we recommend consulting Exhibits 30 and 36 in the RMI report[16].

2.6.2 Case of Green Hydrogen-Coal Chemical Production (modified from[35])

Incorporating "green hydrogen" into chemical production is essential for the coal chemical industry to attain "carbon neutrality." The water gas shift process, used after coal gasification to adjust the carbon-hydrogen ratio for subsequent product synthesis, is a major hydrogen source but also a significant emitter of CO2. Substituting "green hydrogen" in the synthesis gas can substantially decrease carbon emissions in coal-to-chemical production.

Baofeng, a Chinese coal chemical company, has embarked on a 200MW photovoltaic power generation project aimed at producing 160 million cubic meters of green hydrogen annually. This initiative is set to integrate with their existing methanol production, reducing carbon emissions by 240 million tons and cutting down raw coal usage by 105,000 tons. Profitability forecasts suggest that using "green hydrogen" for olefin production is viable, with the benchmark price at 1.34 CNY per cubic meter. Over time, hydrogen costs could drop to around 0.7 CNY per cubic meter, aligning with the cost of hydrogen derived from fossil fuels. By integrating green hydrogen into the coal-to-olefins system, the total CO2 emission reduction is estimated at 67.9%[36].

2.6.3 CCUS Cases in China (modified from [37])

On August 29, 2022, Sinopec launched China's largest carbon capture, utilization, and storage (CCUS) industry chain demonstration base, the "Qilu Petrochemical-Shengli Oilfield Mega-ton CCUS Project," marking China's first mega-ton CCUS project and signifying the industry's advancement into the technology demonstration phase. This project aims to reduce CO2 emissions by 1 million tons annually, equivalent to the absorption capacity of nearly 9 million trees. It plays a crucial role in developing an "artificial carbon cycle" model, offering valuable engineering experiences and technical data for large-scale CCUS projects in China, thus aiding in achieving the "double carbon" goal.

The Qilu Petrochemical-Shengli Oilfield project captures CO2 from Qilu Petrochemical's coal gasification unit in the fertilizer plant, noted for its high-quality, stable, and up to 90% pure emissions. Utilizing liquefaction purification technology,

the project is energy-efficient and cost-effective. The captured CO2 is transported to Shengli Oilfield for oil displacement and storage, integrating capture, displacement, and storage processes. Spanning over 25 million tons of ultra-low permeability reservoir reserves and with 73 injection wells, it's projected to inject over 10 million tons of CO2, boost oil production by nearly 3 million tons, and increase recovery rates by more than 12% over 15 years.

Shengli Oilfield has pioneered the "carbon dioxide high-pressure miscible flooding" technology, injecting 640,000 tons of CO2 and sealing over 500,000 tons, enhancing oil production by more than 100,000 tons. Sinopec's CCUS efforts have been significant, capturing 1.52 million tons of CO2 in 2021 alone. The first CCUS project in a Chinese coal-fired power plant was initiated in 2012 at Shengli Oilfield, developing comprehensive technology and economic evaluation for carbon capture, flooding, and storage. In 2015, Sinopec began recovering CO2 tail gas from synthetic ammonia and coal-to-hydrogen plants for oil field injection, setting a precedent for comprehensive CO2 utilization across its operations.

3. Supplementary Tables

Table S1 National Standards Used in Determining the Uncertainty Range

Product	National Standards
Ethylene	GB 30250-2013
Coal to olefins	GB 30180-2013
Coal to methanol	GB 29436.1-2012
Natural gas to methanol	GB 29436.2-2015
Coke oven gas to methanol	GB 29436.4-2015
Synthetic ammonia	GB 29436.4-2015
Calcium carbide	GB 21343-2015
Ethylene glycol	GB 32048-2015
Coke	GB 21342-2013

In the following tables, the unit of energy consumption is the kilogram of standard oil equivalent per ton (kgoe/t) or kilogram of standard coal equivalent per ton (kgce/t).

Table S2 Energy/Fossil Fuels Consumption Range of Ethylene Production (GB 30250-2013)

Product	Production Process	Unit	Threshold value	Standard value	Advanced value
Ethylene	Large plants (> 300kt/a)	kgoe/t	≤830	≤640	≤610
	Small plants (≤ 300kt/a)		≤720		

Table S3 Energy/Fossil Fuels Consumption Range of Coal to Olefins (GB 30180-2013)

Product	Production Process	Unit	Threshold value	Standard value	Advanced value
Ethylene & Propylene	Coal to Olefins	kgce/t	≤4500	≤4000	≤3700
Propylene	Coal to Propylene		≤6000	≤5500	≤5200

Table S4 Energy/Fossil Fuels Consumption Range of Coal to Methanol (GB 29436.1-2012)

Product	Production Process	Unit	Threshold value	Standard value	Advanced value
Methanol	Lignite to Methanol	kgce/t	≤2400	≤2000	≤1900
	Bituminous coal to Methanol		≤2200	≤1800	≤1700
	Anthracite to Methanol		≤1800	≤1600	≤1500

Table S5 Energy/Fossil Fuels Consumption Range of Natural Gas to Methanol (GB 29436.2-2015)

Product	Production Process	Unit	Threshold value	Standard value	Advanced value
Methanol	Natural Gas to Methanol	kgce/t	≤1460	≤1150	

Table S6 Energy/Fossil Fuels Consumption Range of Coke Oven Gas to Methanol (GB 29436.4-2015)

Product	Production Process	Unit	Threshold value	Standard value	Advanced value
Methanol	Coke Oven Gas to Methanol	kgce/t	≤1650	≤1500	≤1300

Table S7 Energy/Fossil Fuels Consumption Range of Ammonia (GB 29436.4-2015)

Product	Production Process	Unit	Threshold value	Standard value	Advanced value
Ammonia	High-Quality Lump Anthracite to Ammonia	kgce/t	≤1500	≤1350	≤1150
	Lump Anthracite & Briquette to Ammonia		≤1700	≤1550	≤1320
	Pulverized Coal to Ammonia		≤1680	≤1650	≤1500
	Natural Gas to Ammonia		≤1250	≤1100	≤1050

Table S8 Energy/Fossil Fuels Consumption Range of Calcium Carbide Production (GB 21343-2015)

Product	Production Process	Unit	Threshold value	Standard value	Advanced value
Calcium Carbide		kgce/t	≤1000	≤823	

Table S9 Energy/Fossil Fuels Consumption Range of Ethylene Glycol Production (GB 32048-2015)

Product	Production Process	Unit	Threshold value	Standard value	Advanced value
Ethylene Glycol	Ethylene to EG	kgce/t	≤500	≤430	≤230
	Syngas to EG		≤1430	≤1120	≤1045

Table S10 Energy/Fossil Fuels Consumption Range of Coking (GB 21342-2013)

Product	Production Process	Unit	Threshold value	Standard value	Advanced value
Coke	Top-loading coke ovens	kgce/t	≤150	≤122	≤115
	Tamping coke ovens		≤155	≤127	≤115

4. References

1 Chen, J., Qin, Z., Meng, Y. & Zhang, Y. Study on the organic element composition of coal group components. *China Coal* **37**, 85-88 (2013).

2 Dau, J., Qin, S., Tao, S., Zhu, G. & Mi, J. Development trend of natural gas industry in China and important progress of natural gas geological theory. *Natural Gas Geoscience*, 127-142 (2005).

3 Wu, C. Research on the process technology of coke oven gas to methanol. *Gas and Thermal Power*, 36-42 (2008).

4 Kang, W. Coal chemical technology. (Sinopec Press, Beijing, 2017);

5 Coal Science and Technology Research Institute. Compiling Instruction on National Standard: Energy consumption limit per unit product of coal chemical industry. (Coal Science and Technology Research Institute, Beijing, 2020);

6 Gao, J. Acetylene production methods and technological progress. *Natural Gas Chemical*, 63-66 (2005).

7 Nan Jia, Xiaowen Lu, Jinfei Bian & Jingxin Liu. Thermal efficiency and energy-saving technological of industrial coal-fired boilers. *Energy Conservation* **40**, 27-29 (2021).

8 Moretti, C., Moro, A., Edwards, R., Rocco, M. V. & Colombo, E. Analysis of standard and innovative methods for allocating upstream and refinery GHG emissions to oil products. *Applied Energy* **206**, 372-381 (2017).

9 Jing, L. *et al.* Carbon intensity of global crude oil refining and mitigation potential. *Nature Climate Change* **10**, 526-532 (2020).

10 McMurray, A., Pearson, T. & Casarim, F. Guidance on applying the Monte Carlo approach to uncertainty analyses in forestry and greenhouse gas accounting. (Winrock International, Arlington, VA, USA, 2017); https://winrock.org/wp-content/uploads/2018/03/UncertaintyReport-12.26.17.pdf

11 China National Petroleum & Chemical Planning Institute. Low-carbon development report for petrochemical and chemical industry. (China National Petroleum & Chemical Planning Institute, Beijing, 2022); http://xn--fiqu8gjwaf8ivwklv2e.com/UpLoadFile/files/2022/9/28/01%E6%9D%8E%E5%BF%97%E5%9D%9A-%E7%9F%B3%E5%8C%96%E5%8C%96%E5%B7%A5%E8%A1%8C%E4%B8%9A%E4%BD%8E%E7%A2%B3%E5%8F%91%E5%B1%95%E6%8A%A5%E5%91%8A.pdf

12 China National Petroleum & Chemical Planning Institute. Low-carbon development of raw materials in petrochemical and chemical industry. (China National Petroleum & Chemical Planning Institute, Beijing, 2022); http://xn--fiqu8gjwaf8ivwklv2e.com/UpLoadFile/files/2022/9/28/02%E9%BE%9A%E5%8D%8E%E4%BF%8A-%E7%9F%B3%E5%8C%96%E5%8C%96%E5%B7%A5%E8%A1%8C%E4%B8%9A%E5%8E%9F%E6%96%99%E4%BD%8E%E7%A2%B3%E5%8C%96%E5%8F%91%E5%B1%95%E7%A0%94%E7%A9%B6.pdf

13 China National Petroleum & Chemical Planning Institute. Low-carbon development technologies in the petrochemical and chemical industry. (China National Petroleum & Chemical Planning Institute, Beijing, 2022); http://xn--fiqu8gjwaf8ivwklv2e.com/UpLoadFile/files/2022/9/28/03%E6%9C%B1%E5%BD%AC%E5%BD%AC-%E7%9F%B3%E5%8C%96%E5%8C%96%E5%B7%A5%E8%A1%8C%E4%B8%9A%E4%BD%8E%E7%A2%B3%E6%8A%80%E6%9C%AF%E7%A0%94%E7%A9%B6.pdf

14 China National Petroleum & Chemical Planning Institute. Carbon emission coefficient of petrochemical and chemical products. (China National Petroleum & Chemical Planning Institute, Beijing, 2022); http://xn--fiqu8gjwaf8ivwklv2e.com/UpLoadFile/files/2022/9/28/06%E4%BC%8D%E6%A1%82%E6%9D%BE-%E7%9F%B3%E5%8C%96%E5%8C%96%E5%B7%A5%E4%BA%A7%E5%

93%81%E7%A2%B3%E6%8E%92%E6%94%BE%E7%B3%BB%E6%95%B0%E7%A0%94%E7%A9%B6.pdf
15 McKinsey & Company. China Accelerates the Move towards Carbon Neutrality (Coal Chemical Industry Chapter): Carbon Emission Reduction Pathways for the Coal Chemical Industry. (McKinsey & Company, Shanghai, 2021); https://www.mckinsey.com.cn/%E4%B8%AD%E5%9B%BD%E5%8A%A0%E9%80%9F%E8%BF%88%E5%90%91%E7%A2%B3%E4%B8%AD%E5%92%8C%E7%85%A4%E5%8C%96%E5%B7%A5%E7%AF%87%EF%BC%9A%E7%85%A4%E5%8C%96%E5%B7%A5%E8%A1%8C%E4%B8%9A%E7%A2%B3/
16 Rocky Mountain Institute. Transforming China's Chemicals Industry: Pathways and Outlook under the Carbon Neutrality Goal. (RMI, Beijing, 2022); https://rmi.org/insight/transforming-chinas-chemicals-industry/
17 Wang, M. Performance analysis and suggestions on hydrogen energy coupling coal chemical system (in Chinese). *Modern Chemical Industry* **41**, 4-8 (2021).
18 Meng, F. *et al.* Planet-compatible pathways for transitioning the chemical industry. *Proceedings of the National Academy of Sciences* **120**, e2218294120 (2023).
19 Ministry of Ecology and Environment of China. Key tasks related to the management of corporate greenhouse gas emission reports in 2022 (Ministry of Ecology and Environment of People's Republic of China, Beijing, 2022); https://www.mee.gov.cn/xxgk2018/xxgk/xxgk06/202203/t20220315_971468.html
20 Gabrielli, P., Gazzani, M. & Mazzotti, M. The Role of Carbon Capture and Utilization, Carbon Capture and Storage, and Biomass to Enable a Net-Zero-CO2 Emissions Chemical Industry. *Industrial & Engineering Chemistry Research* **59**, 7033-7045 (2020).
21 Zhang, X., Yang, X. & Lu, X. China Carbon Dioxide Capture Utilization and Storage (CCUS) Annual Report (2023). (China 21 Century Yicheng Management Center, Global CCS Institute, Tsinghua University, Beijing, 2023); https://www.globalccsinstitute.com/wp-content/uploads/2023/03/CCS-Progress-in-China-CN.pdf
22 Wei, Y. *et al.* Roadmap for Achieving China's Carbon Peak and Carbon Neutrality Pathway (in Chinese). *Journal of Beijing Institute of Technology(Social Sciences Edition)* **24**, 13-26 (2022).
23 Keller, F., Mamani Soliz, P., Seidl, L. G., Lee, R. P. & Meyer, B. Life cycle inventory data generation by process simulation for conventional, feedstock recycling and power-to-X technologies for base chemical production. *Data in Brief* **41**, 107848 (2022).
24 Yang, X., Nielsen, C. P., Song, S. & McElroy, M. B. Breaking the hard-to-abate bottleneck in China's path to carbon neutrality with clean hydrogen. *Nature Energy* **7**, 955-965 (2022).
25 Tu, Q.-h. Current situation and development trend of China's aromatics industry (in Chinese). *Chemical Industry*, 36-44 (2020).
26 SinoCarbon and Energy Foundation. Research on the Transformation Development Plan of China's Coal Industry under the dual carbon goals (SinoCarbon and Energy Foundations, Beijing, 2022); https://www.efchina.org/Attachments/Report/report-lceg-20220710/%E5%8F%8C%E7%A2%B3%E7%9B%AE%E6%A0%87%E4%B8%8B%E7%85%A4%E7%82%AD%E8%A1%8C%E4%B8%9A%E8%BD%AC%E5%9E%8B%E5%8F%91%E5%B1%95%E7%A0%94%E7%A9%B6%E6%8A%A5%E5%91%8A.pdf
27 Xinhua News. The world's first mercury-free production technology for carbide-based polyvinyl chloride (PVC) is released. (Xinhua News, Ordos 2023); https://www.xinhuanet.com/energy/20230220/468b29ee619b4f1094d1ef66d39f5efc/c.html
28 Lange, J.-P. Towards circular carbo-chemicals–the metamorphosis of petrochemicals.

Energy & Environmental Science (2021).
29 Wolfersdorf, C., Forman, C., Keller, F., Gootz, M. & Meyer, B. CO2-to-X and Coal-to-X Concepts in Pulverized Coal Combustion Power Plants. *Energy Procedia* **114**, 7171-7185 (2017).
30 Kätelhön, A., Meys, R., Deutz, S., Suh, S. & Bardow, A. Climate change mitigation potential of carbon capture and utilization in the chemical industry. *Proceedings of the National Academy of Sciences of the United States of America* **116**, 11187 (2019).
31 Davis, S. J. *et al.* Net-zero emissions energy systems. *Science* **360**, eaas9793 (2018).
32 Keller, F., Voss, R. L., Lee, R. P. & Meyer, B. Life cycle assessment of global warming potential of feedstock recycling technologies: Case study of waste gasification and pyrolysis in an integrated inventory model for waste treatment and chemical production in Germany. *Resources, Conservation and Recycling* **179**, 106106 (2022).
33 Jiang, X. *et al.* Assessment of Plastic Stocks and Flows in China: 1978-2017. *Resources, Conservation and Recycling* **161**, 104969 (2020).
34 Klotz, M., Haupt, M. & Hellweg, S. Limited utilization options for secondary plastics may restrict their circularity. *Waste Management* **141**, 251-270 (2022).
35 CITIC Securties. Pioneer in "Green Hydrogen" Manufacturing, Building a Carbon-Neutral Coal Chemical Leader. Baofeng Energy In-depth Tracking Report. (CITIC Securties, Beijing, 2021);
36 Guosen Securties. Coal-to-olefins coupled with green hydrogen, carbon-neutral layout of coal-based leaders. (Guosen Securties, Shenzhen, 2022); https://pdf.dfcfw.com/pdf/H3_AP202201051538608785_1.pdf?1641395887000.pdf
37 National Energy Administration. China's first million-ton CCUS project fully completed and put into operation. (National Energy Administration, Beijing, 2022); http://www.nea.gov.cn/2022-09/02/c_1310658658.htm
