# Supplementary material for: Tracing fossil-based plastics, chemicals and fertilizers production in China
Source: Nat Commun. 2024 May 8;15:3854. doi: 10.1038/s41467-024-47930-0 (PMC11078955; doi:10.1038/s41467-024-47930-0)
Supplement: Supplementary file 3 — Description of Additional Supplementary Files [file 41467_2024_47930_MOESM3_ESM.pdf]

Description of Additional Supplementary Files

File Name: Supplementary Data 1
Description: Figure: It provides the original data of Figures 1-5.

File Name: Supplementary Data 2
Description: Flows: This section provides calculation data across six key areas: the division of energy and feedstock used in fossil hydrocarbons, linking primary chemicals to downstream production, tracing the carbon source, evaluating CO2 mitigation potential, and comparing emission intensities. Uncertainty ranges are indicated alongside the process-based coefficients. All references and data sources are documented in the accompanying spreadsheet.
